# Interpretation of C-Reactive Protein Concentrations in Critically Ill Patients

**DOI:** 10.1155/2013/124021

**Published:** 2013-10-28

**Authors:** Christophe Lelubre, Sophie Anselin, Karim Zouaoui Boudjeltia, Patrick Biston, Michaël Piagnerelli

**Affiliations:** ^1^Department of Intensive Care, CHU-Charleroi, Université Libre de Bruxelles, 92 Boulevard Janson, 6000 Charleroi, Belgium; ^2^Experimental Medicine Laboratory, CHU-Charleroi, ULB 222 Unit, 6110 Montigny-Le-Tilleul, Belgium

## Abstract

Infection is often difficult to recognize in critically ill patients because of the marked coexisting inflammatory process. Lack of early recognition prevents timely resuscitation and effective antimicrobial therapy, resulting in increased morbidity and mortality. Measurement of a biomarker, such as C-reactive protein (CRP) concentration, in addition to history and physical signs, could facilitate diagnosis. Although frequently measured in clinical practice, few studies have reported on the pathophysiological role of this biomarker and its predictive value in critically ill patients. In this review, we discuss the pathophysiological role of CRP and its potential interpretation in the inflammatory processes observed in critically ill patients.

## 1. Introduction

Sepsis, defined as an intense immune reaction occurring as a result of the presence of a pathogen in the organism, is frequent in critically ill patients and is associated with high morbidity and mortality [1, 2]. Recognition and early therapy are the cornerstones of management [3, 4]. When the diagnostic probability of sepsis is high (e.g., a patient with bacteremia complicated by shock and multiple organ failure), measurement of a biological marker (a biomarker) is not really necessary before starting appropriate treatment. However, when a diagnosis of sepsis is less obvious, biomarkers may be more relevant, especially if measurement of the biomarker is rapid and cheap, and results have high specificity and sensitivity for sepsis. Of course, the biomarker result should not be the only trigger for a decision to treat or not, but should be combined with the presence of clinical signs suggesting infection [5]. In this setting, plasma C-reactive protein (CRP) measurements are frequently used as a biomarker.

In this paper, we will briefly review the biochemistry of CRP and its known (patho) physiological roles. As a biomarker for critically ill patients, we discuss its value at intensive care (ICU) admission and its time course during inflammatory states, such as those observed in septic patients. Finally, we describe its pertinence in two particular types of infection: pneumonia and hepatic failure. The role of CRP as a biomarker for cardiovascular disease is not described here and was reviewed recently [6]. 

## 2. Biochemical Characteristics of CRP

As a serum amyloid P component, CRP belongs to the pentraxin family of calcium-dependent ligand-binding plasma proteins. This family is highly conserved during evolution. 

Human CRP is composed of five identical, nonglycosylated polypeptide subunits, each composed of 206 amino acid residues [7]. These subunits are noncovalently associated in an annular configuration with cyclic pentameric symmetry. The binding site of CRP is composed of subunits with 2 calcium ions, located on the concave face of the protein [7]. 

Recently, monomeric CRP, resulting from the loss of its pentameric symmetry, has been described; this form probably has greater prothrombotic properties [8]. 

## 3. Synthesis of CRP

CRP was first discovered by Tillet and Francis in 1930 in the sera of patients with *Streptococcus pneumoniae* pneumonia and was called the “fraction C protein” [9]. At that time, CRP was considered as a marker of infection because the onset of the precipitation reaction observed with the sera of these patients was largest when they were critically ill [9]. 

In healthy Caucasian volunteers, median serum concentrations of CRP, as assessed by solid phase radioimmunoassay, were reported to be 0.8 mg/L (interquartiles 0.34 to 1.7 mg/L) [10], although some of the differences in reference values may have been related to ethnic subgroups [11]. Approximately 50% of the individual variation in physiologic CRP concentrations is genetically attributable to noncoding polymorphisms in the CRP gene, located on chromosome 1.

CRP is a positive acute phase protein produced by the liver in response to stimulation by interleukin (IL)-6. Serum concentrations can increase by up to 1000-fold in inflammation, as compared to physiologic concentrations. Whereas proinflammatory cytokines (e.g., IL-1, IL-6, and tumor necrosis factor *α*-[TNF-*α*]) appear within one hour after the start of bacterial infection, and procalcitonin (PCT) after 5 hours, the hepatic synthesis of CRP starts 6 to 8 hours after onset [12] and peak concentrations are reached between 36 to 50 hours after infection has started. The half-life of CRP is 19 hours [13] and it is cleared by the liver. Although extrahepatic CRP synthesis has been reported in neurons [14], atherosclerotic plaques [15], lymphocytes [16], and adipocytes [17], this synthesis has very little impact on serum concentrations. This local production of CRP may be a process of localized inflammation and a marker for local cellular damage [14, 17].

## 4. Physiologic Roles of CRP

**Figure 1:**
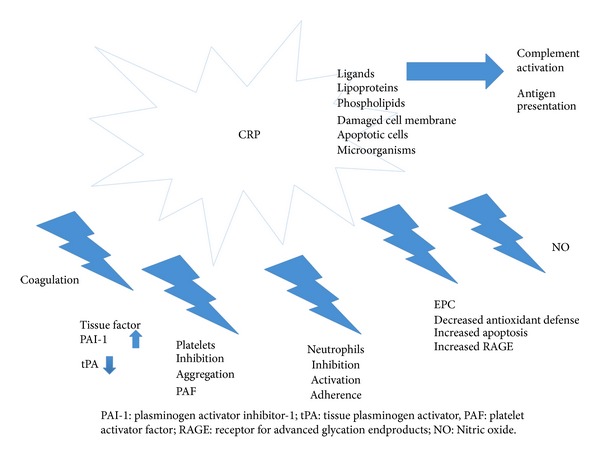
Summary of the principal physiological roles of C-reactive protein.

The physiological roles of CRP are summarized in Figure 1. Even though CRP has been known for more than 80 years [9], its exact physiological roles remain largely unknown. Before describing current knowledge regarding the anti- and proinflammatory roles of CRP, it is important to remember that it may not always be possible to directly extrapolate results obtained in animal models to the clinical situation in humans. Indeed, although CRP has been highly conserved during evolution, the time course of CRP synthesis is related to the species. For example, in mice, the most frequently used animal model, CRP concentrations only increase slightly during the acute phase response. Moreover, considerable variations between species are observed with respect to ligand-binding specificity and glycosylation status [7]. 

### 4.1. Activation of the Complement Pathway

Globally, the biological role of CRP after binding to ligands is to trigger the complement pathway [7] CRP binds with greatest affinity to phosphocholine residues, and with less affinity to native and modified lipoproteins [44], damaged cell membranes, a number of phospholipids, small nuclear ribonucleoprotein particles, and apoptotic cells [45]. It also binds constituents of microorganisms (compounds of the membranes of bacteria, fungi, parasites, and plants) and facilitates antigen presentation on dendritic cells [46]. When aggregated or bound to macromolecular ligands, CRP is recognized by complement protein C1q and potently activates the classical complement pathway, engaging C3, the main molecule of adhesion of the complement system, and the terminal membrane attack complex (C5–C9) [47]. Bound CRP may also provide secondary binding sites for factor H and, thereby, regulate alternative-pathway amplification and C5 convertase [48, 49]. 

### 4.2. Antiinflammatory Effects of CRP

*In vitro*, the antiinflammatory effects of CRP are the result of inhibition of neutrophil activation, adherence, and trafficking into tissues [50]. CRP may decrease the production of cytokines and the expression of adhesion molecules (intercellular adhesion molecule (ICAM) 1, E and P selectins) [51]. CRP inhibits phospholipases and platelet-activating factor (PAF), protecting the platelet membrane and decreasing the aggregation process [8].

*In vivo*, the antiinflammatory effects are illustrated by the study of Xia and Samols [50] in which mortality rates in transgenic mice overexpressing CRP (CRP concentrations between 75–200 mcg/mL) decreased after injection of lipopolysaccharide (LPS) or other proinflammatory mediators, such as PAF or TNF-*α*, compared to CRP-deficient mice (CRP concentrations < 20 mcg/mL) [50]. 

### 4.3. Proinflammatory Effects of CRP

CRP also has a proinflammatory role. When recombinant CRP is incubated with endothelial cells from the aorta, multiple genes for proinflammatory proteins, such as IL-8, fibronectin, and plasminogen activator inhibitor 1 (PAI-1), are expressed [52]. These results must be interpreted with regard to the *in vitro* model and the type of endothelial cells studied [52]. In a mouse model, Hirschfield et al. [53] observed no protective effects of recombinant human CRP injection after LPS challenge, in contrast to the study by Xia and Samols [50]. The contrasting results of these two studies [50, 53] highlight the difficulties associated with extrapolating results from one model to another.

### 4.4. CRP in Coagulation and Fibrinolysis

CRP may be the link between inflammation and coagulation in sepsis [13]. Indeed, CRP contributes to a prothrombotic state by the liberation of tissue factor (TF) by monocytes [54], endothelial cells, and smooth skeletal muscle cells [55]. The *in vitro* study by Wang et al. [52] showed increased expression of the gene for PAI-1 in cells incubated with CRP, and decreased activity of tissue plasminogen activator (tPA) has also been observed [55]. All of these effects may lead to an imbalance in the fibrinolytic system [56]. A study performed in healthy volunteers confirmed these results [57]; after injection of recombinant CRP, there was an increase in fibrinolysis, as suggested by increased concentrations of the prothrombotic fragments, F1 + F2, and D-dimers. Identical results were observed in hypercholesterolemic patients [58]. However, these studies demonstrating procoagulant effects of CRP have been criticized due to the possibility of endotoxin contamination and need confirmation [59]. Nevertheless, in 32 critically ill patients with and without sepsis, we observed a significant correlation (*r* ^2^ = 0.45, *P* < 0.001) between CRP concentrations at ICU admission and fibrinolysis assessed by the euglobulin lysis test [60].

### 4.5. CRP and the Nitric Oxide (NO) Pathway

CRP may also modulate NO bioavailability and NO synthase (NOS) expression. This effect on NO production remains controversial due to the different models studied (animals or *in vitro*). In transgenic mice expressing human CRP, Grad et al. [61] observed that NOS and NO expressions were locally and systematically suppressed after arterial femoral injury. In contrast, Clapp et al. [62] observed increased NO reactivity with no change in NOS activity after incubation of rat aorta with purified CRP. The observed vasodilatory effect was not due to the NO itself but more likely the result of increased expression of GTP cyclohydrolase-1, the rate-limiting enzyme in the synthesis of tetrahydrobiopterin, the NOS cofactor [62]. In several studies involving endothelial progenitor cells, CRP was shown to decrease endothelial NO production, decrease antioxidant defenses of the cells, increase expression of the receptor for advanced glycation end-products (RAGE), and induce apoptosis of endothelial cells [63, 64].

## 5. CRP Concentrations as a Biomarker of Infection in Septic Patients

Sepsis remains an important cause of mortality in the ICU [1, 3, 4] and delay in appropriate antibiotherapy may increase morbidity and mortality in septic patients [65]. Because of wide availability, good reproductibility, and low cost, CRP concentrations could be an attractive biomarker [66]. Questions remain regarding the predictive value, sensitivity, and specificity of CRP to diagnose infection in ICU patients, especially in patients receiving specific treatments (e.g., glucocorticoids [67, 68] or statins [69]). The various studies performed in ICU patients are summarized in Table 1. Ugarte et al. [18] measured CRP and PCT concentrations in 180 critically ill patients with (*n* = 111) and without infection (*n* = 79). Surgical patients and patients in whom there was a doubt regarding the presence of infection (antibiotherapy without bacteriological proof) were excluded. The median CRP value was significantly higher in infected patients (12.1 versus 5.6 mg/dL), with a best cut-off value of 7.9 mg/dL. However, on admission, 33% of the noninfected patients had CRP concentrations greater than 7.9 mg/dL, making it difficult to discriminate patients with and without infection based on this CRP measurement. Similarly, in 74 ICU patients, Reny et al. [19] observed that CRP values were more elevated in patients with proven infection (*n* = 28) compared to those without (191 ± 123 versus 83 ± 91 mg/L, *P* < 0.0001). No threshold for CRP was identified to discriminate between infected and noninfected patients. The change in CRP concentration between admission and day 4 was the best predictor for recovery [19].

Póvoa et al. [20] also studied CRP concentrations at ICU admission as a marker of infection. In 112 patients, these authors observed a significantly higher CRP concentration in infected (*n* = 76) versus noninfected patients (*n* = 36). CRP values correlated well with the severity of the infection. For a cut-off of 8.7 mg/dL, the sensitivity and specificity of CRP for a diagnosis of infection were 93.4 and 86.1%, respectively. The specificity increased to 100% if CRP was combined with a temperature >38.2°C. Regrettably, the protocol of this study is difficult to transpose to the clinical situation. Indeed, the CRP concentrations included in the study for the nonseptic group were the values measured after 2 days of ICU stay and were compared to the leukocyte count and temperature on the day of ICU admission [20]. The same group [21] validated this CRP cut-off as a predictor of infection in a prospective study. They studied a very limited number of infected patients (*n* = 35) because only patients with a delay of 5 days between positive microbiological cultures and start of antibiotics were included. Patients with this cut-off of 8.7 mg/dL associated with a daily variation in CRP >4.1 mg/dL had an 88% risk of infection [21].

In a prospective monocenter study, Lobo et al. [22] stratified 303 consecutive patients admitted to the ICU for a minimum of 48 hours according to the CRP concentration at ICU admission (<1, between 1–10 and >10 mg/dL). They observed that CRP concentration at ICU admission was associated with organ dysfunction, ICU length of stay, and mortality. A CRP concentration >10 mg/dL was associated with proven infection in 73% of the patients as compared to 31% when the CRP was <1 mg/dL [22]. The time course of CRP concentrations also provided some interesting insights in the patients with high CRP concentrations (>10 mg/dL). A decreasing concentration in the first 48 hours was associated with a mortality of 15.4%, whereas mortality reached 60.9% for patients in whom the CRP concentration increased (RR 0.25, CI: 0.07–0.97; *P* < 0.05) [22]. A study by Castelli et al. [23] provided similar results, but the results were not confirmed in a study by Silvestre et al. [24] In the study by Castelli et al. [23], the authors compared CRP concentrations in 255 patients (111 septic, 49 trauma, 45 with and 50 without systemic inflammatory response syndrome [SIRS]) observed for a total of 1826 days [23]. The best cut-off value of CRP concentration for diagnosis of sepsis was 12.8 mg/dL, and these authors also reported a delay of 48 hours to reach the maximum CRP value. In the study by Silvestre et al. [24], which included 158 septic patients, the authors observed no significant differences in CRP concentrations at ICU admission between survivors and nonsurvivors (25.3 ± 13.7 versus 28.2 ± 13.2 mg/dL), although the SOFA score was higher in nonsurvivors (Sequential Organ Failure Assessment-SOFA-Score [70]: 11 ± 4 versus 7 ± 3, *P* < 0.001). Moreover, the ICU mortality rates of septic patients with CRP concentrations <10, 10–20, 20–30, 30–40 and >40 mg/dL were 20%, 34%, 30.8%, 42.3%, and 39.1%, respectively, *P* = 0.7, and the area under the curve for CRP to diagnose an infection was 0.55 (0.45–0.65), no better than leukocyte count or temperature. Restricting the analysis to include only patients with microbiological proof of infection did not alter these findings [24]. 

Confirming that the time course of CRP concentrations is more important than a single admission value, Póvoa et al. [25] observed no significant differences between CRP in survivors and nonsurvivors until day 2 of antibiotic therapy in a multicenter prospective observational study including 891 septic patients. On the subsequent three days, the CRP concentration in survivors was significantly lower (*P* < 0.001) than that of nonsurvivors. After adjusting for the Simplified Acute Physiology Score II and severity of sepsis, CRP time course was significantly associated with ICU mortality (OR = 1.03, CI 95% 1.02–1.04, *P* < 0.001). The hospital mortality rates of patients with fast response, slow response, and no response patterns were 23, 30, and 41%, respectively, *P* = 0.001. Nonresponders had a significant increase in the odds of death (OR = 2.5, CI 95% 1.6–4.0, *P* < 0.001) when compared with fast responders [25]. 

Two studies specifically reported a relationship between bacteremia and CRP concentrations (Table 1). Vandijck et al. [26] observed a relationship between the presence of Gram-negative bacteremia in 48 ICU patients and variation in CRP concentrations of 5 mg/dL between 2 days prior to and the day after the onset of bacteremia [26]. Also in bacteremia, Póvoa et al. [27] suggested the importance of daily measurement of CRP concentrations in the assessment of appropriate antibiotherapy. Although body temperature and leukocyte counts were not significantly different in survivors and nonsurvivors in 44 patients with bacteremia, the ratio of CRP concentrations on the day measured and at admission did not change in nonsurvivors in contrast to survivors in whom changes were observed already at day 2. Nevertheless, it was necessary to wait until day 4 to observe a relationship of the ratio with outcome [27]. This observation was also suggested in the meta-analysis of Zhang and Ni [28]. This meta-analysis included 1969 patients from 14 studies with large heterogeneity (*I* ^2^ = 92%) and showed that the variation in CRP could be associated with mortality only after 48 hours. 

CRP concentrations can also be used as a marker of infection in neutropenic patients. Póvoa et al. [29] compared body temperature, leukocyte count, and CRP concentrations in septic neutropenic and nonneutropenic patients admitted to the ICU (Table 1). There were no differences in core temperature between the 2 groups at admission, but higher concentrations of CRP were observed in neutropenic than in nonneutropenic patients. Among the neutropenic patients, CRP concentrations at ICU admission were not influenced by the severity of neutropenia. Nevertheless, white blood cell count was weakly correlated with CRP for all patients (*r* = 0.25, *P* = 0.012). However, although severity scores and mortality rates were similar among groups, infection site and use of specific treatments (e.g., noninvasive ventilation) were different, making interpretation of the results difficult [29]. 

In a limited number of patients, Fraunberger et al. [30] looked at the predictive value of CRP, PCT, and IL-6 for development of sepsis after the first incidence of fever in 38 critically ill patients. They observed no differences in CRP concentrations at the onset of fever between survivors and nonsurvivors and CRP concentrations had the lowest AUC for discriminating infection compared to the two other markers. Nevertheless, CRP concentrations were compared to those in volunteers and not to values before fever onset or at ICU admission. Moreover, few data about patient characteristics (e.g., length of the inflammatory process) were reported in the paper [30]. Recently, Su et al. [31] compared values of CRP, PCT, and soluble triggering receptor expressed on myeloid cells (sTREM)-1 at ICU admission and during a new fever episode in the first 48 hours of the ICU stay. As expected, all these biomarkers were more elevated at ICU admission in septic patients compared to patients with SIRS. The authors observed that only the CRP level at the onset of fever could discriminate patients with or without bacteremia [31]. Although CRP changes in fever seemed to be interesting to determine new onset infection, CRP concentration was significantly lower in patients with bacteremia than in those without (9.6 ± 6.5 versus 13.2 ± 8 mg/dL, *P* = 0.03) [31]. 

The time course of CRP as a marker of appropriate treatment, as has been suggested for PCT [32, 71], and as a marker for the end of infection is of potential interest, although few studies have reported these aspects during sepsis. Schmit and Vincent [72] reported the time course of CRP in 50 septic patients with adequate (*n* = 24) or inadequate (*n* = 18) empiric antibiotherapy and in surgical patients who needed reoperation for uncontrolled infection (*n* = 8) [72]. As expected, CRP concentrations decreased faster during the first 48 hours when the antibiotherapy was adequate, but an increase in CRP concentration of a minimum of 2.2 mg/dL over the 48-hour period was predictive of inadequate antibiotherapy with a sensitivity of 77% and a specificity of 67% [72]. These results were identical if a longer delay between CRP measurements was used. The take-home message of this study was the need for at least two CRP measurements with a delay of 48 hours to estimate the appropriateness of antibiotherapy, as suggested by the meta-analysis from Zhang and Ni [28]. Another interesting aspect of this study was the time course of CRP in a surgical population with uncontrolled infection. Regrettably, the number of patients studied was limited (*n* = 8), and the delay for reoperation in case of uncontrolled infections was not reported, limiting the conclusions that can be drawn regarding the usefulness of CRP values in this particular population [72]. Further studies looking at the time course of CRP in relation to the need for reoperation and outcome are needed. Comparison of the time course of CRP values in relation to etiologies of sepsis (e.g., peritonitis versus pneumonia) may also be interesting. 

In summary, diagnosis of infection in ICU patients remains difficult. One CRP value is probably not sufficient to discriminate infected from noninfected patients. The CRP ratio, already at day 2 but certainly at day 4, is more predictive of infection and/or adequate antibiotherapy than individual values. 

### 5.1. CRP Concentrations in Pneumonia

These studies included patients with ventilator-associated pneumonia (VAP) or community-acquired pneumonia (CAP) requiring ICU admission (Table 2). A majority of these patients received specific treatments for their condition, notably corticosteroids, and the effects of these agents on CRP are controversial [67, 68]. In a subgroup of 48 patients with VAP, Póvoa et al. reported higher CRP concentrations but also core temperatures in infected than in noninfected patients. In contrast, leukocyte counts were not different [19]. No relationship with mortality was reported. Regrettably, the CRP difference between patients with VAP and other septic patients was not calculated [19]. In another study that included 45 patients with VAP, Hillas et al. [35] observed no difference in CRP concentrations at VAP diagnosis between survivors and nonsurvivors, but an increase in CRP between days 1 and 7 increased the risk of developing septic shock. Nevertheless, an important issue when trying to use these results in the clinical situation is the long delay (7 days) needed to discriminate between survivors and nonsurvivors; indeed, it would be difficult to wait such a long time before adapting therapy (e.g., changing antibiotherapy) [35]. 

Seligman et al. [36] also reported no difference in admission CRP concentrations between survivors and nonsurvivors in patients with VAP (*P* = 0.77), but in a logistic regression model, a decrease in CRP between day 4 and day 0 was associated with a favorable outcome (odds ratio 7.4 (95% CI: 1.58–34.73). These correlations were also found for procalcitonin [36]. Similarly, Póvoa et al. [37] noted the importance of delta CRP between day 4 and admission in 47 patients with VAP. By day 4, a CRP of 0.6 times the initial level was a marker of poor outcome (sensitivity 0.92; specificity 0.59). All patients with fast and slow CRP concentration response patterns survived, whereas those showing no response and a biphasic response pattern exhibited a mortality of 78 and 75%, respectively [37]. 

In two studies, one retrospective and monocenter [38] and the second prospective and multicenter [39], Coelho et al. investigated the time course of CRP concentrations in ICU patients admitted for CAP. These authors defined several patterns in relation to the time course of CRP between the value at admission and that at days 5 and 7. Again, although CRP concentrations were not different between survivors and nonsurvivors at ICU admission, the CRP ratio (Day 7/Day1) decreased significantly more rapidly in survivors. Already at day 5, a CRP of more than 0.5 mg/dL above the baseline value was associated with a poor outcome. Mortality rates for all patients were correlated to the CRP patterns described [38]. In the largest study [39], the authors specifically analyzed the subgroup of mechanically ventilated patients (*n* = 111) and also showed different patterns of CRP between survivors and nonsurvivors.

In contrast, Bajwa et al. [40], observed a lower CRP concentration in nonsurvivors (*n* = 70, 176.5 mg/L (IQR, 173.0)) compared to survivors (*n* = 107, median 133.5 mg/L, IQR, 161.0; *P* = 0.02) in critically ill patients with acute respiratory distress syndrome/acute lung injury (ARDS/ALI). This difference in CRP was observed in patients with pneumonia but not in trauma patients [40]. For these authors, the results suggested a protective role of CRP by inhibiting neutrophil chemotaxis or modulating vascular permeability. Nevertheless, these results may be limited by the fact that nonsurvivors had greater APACHE III scores, were older, and included more cirrhotic patients who perhaps were less able to synthesis CRP [40]. 

CRP may also be a marker of bacterial load and appropriate antibiotherapy in these patients. Indeed, Lisboa et al. [41] investigated the correlation at days 1 and 4 between quantitative tracheal aspirate and CRP concentrations in 68 patients with VAP. They observed a good correlation between the first bacterial load and CRP concentrations (*r* ^2^ = 0.46, *P* < 0.05) but also between variations in bacterial load and CRP over time (*r* ^2^ = 0.59, *P* < 0.05). A CRP ratio of 0.8 at 96 hours seemed to be a useful indicator of adequate antibiotherapy [41]. In less severely ill patients admitted for CAP, Bruns et al. [42] (Table 2) also observed this relationship between the time course of CRP and appropriate empiric antibiotherapy.

Recently, Menendez et al. [43] reviewed the values of several inflammatory biomarkers (CRP, procalcitonin, TNF-*α*, IL-1*β*, IL-6, IL-8, IL-10) in relation to the microorganisms responsible for CAP. CRP levels were higher in patients with CAP with an isolated microorganism than those without (18.10 (9.70–27.30) versus 13.70 (6.95–21.85) mg/dL, *P* = 0.002). CRP concentrations were significantly higher if CAP was associated with bacteremia (23.3 (14.9–35.1) versus 16.1 (8.8–24.1) mg/dL). In contrast, there were no major differences in CRP concentrations in relation to the type of causal microorganism (atypical pathogen, viruses, Gram-positive cocci, and Gram-negative bacilli) [43]. 

### 5.2. CRP Concentrations in Patients with Hepatic Failure

Because CRP is synthesized in the liver, it may be interesting to study its production in hepatic failure, a frequently observed disease in ICU patients, which shares some clinical aspects with the septic process [73]. Bota et al. compared CRP and PCT concentrations in 864 patients with (*n* = 79) and without cirrhosis (*n* = 785) [33]. CRP concentrations were higher in cirrhotic patients with infection compared to cirrhotic patients without infection. These authors did not observe any difference related to the severity of the cirrhosis as assessed by the Child-Pugh classification [33]. However, CRP concentrations in relation to the severity of sepsis (SOFA, vasopressor dosage, PaO_2_/FiO_2_, renal replacement therapy) for each level of cirrhosis were not reported [33]. In other words, could a cirrhotic patient with Child-Pugh C cirrhosis increase CRP concentrations in the same way as a Child Pugh A cirrhotic patient for the same level of sepsis severity? Probably not, and this suggestion is perhaps supported by results from a study by Silvestre et al. [34] in 7 patients admitted for hepatic failure with sepsis. The authors reported very low concentrations of CRP despite proven infection and suggested choosing a biomarker other than CRP in these particular patients. 

## 6. Conclusion

In conclusion, the diagnosis of infection is based on a set of factors, including clinical history, semiology, and clinical and hemodynamic parameters. CRP concentration, with its rapid and cheap measurement, may be a good partner to refine the diagnosis of infection. The time course of CRP concentrations, already at 48 hours, but more interesting at day 4, is of more use than a single measure. Despite daily measurement of CRP in ICUs worldwide, data are relatively limited and studies with more patients, looking at the time course in relation to the etiologies of infection, to the severity and to treatment effects (e.g., of glucocorticoids or statins) are needed to confirm the usefulness of CRP to discriminate infected from noninfected critically ill patients. 

## Figures and Tables

**Table 1:** Summary of the studies in critically ill patients.

Studies	Types of patients included	Conclusions	Remarks
Ugarte et al. [18]	180 patients with (*n* = 111) and without infections (*n* = 79).	Best cut-off value for CRP levels for diagnosis of infection was 7.9 mg/dL	Exclusion of surgical patients

Reny et al. [19]	74 patients with 28 with proven infection	Higher CRP concentrations in infected patients	(i) No cut-off value for CRP (ii) Evolution of CRP between admission and day 4 was related to outcome

Póvoa et al. [20]	Subgroup of patients with VAP (*n* = 48)	Higher CRP levels in patients with VAP than in noninfected patients.	No CRP comparisons between patients with VAP and other infections

Póvoa et al. [21]	Patients with a length of stay ≥3 days	A maximum daily variation of 4.1 mg/dL is a good marker of infection	Long delay between positive culture and start of antibiotics

Lobo et al. [22]	303 patients with a length of stay ≥2 days	(i) High CRP at admission was associated with higher risk of infection (ii) Daily increase in CRP was associated with mortality	Results only applicable if CRP at admission is >10 mg/dL

Castelli et al. [23]	255 patients (111 septic, 49 trauma, 45 with, and 50 without SIRS)	(i) Cut-off for infection: 128 mg/L (ii) Higher values in relation to the severity of sepsis	Maximum CRP with a delay of 24 or 48 hours

Silvestre et al. [24]	158 ICU patients	No relationship between CRP at ICU admission and infection and mortality	No relationship between CRP and presence of a microorganism

Póvoa et al. [25]	891 patients admitted in ICU with diagnosis of community-acquired sepsis. Follow-up of 5 days	(i) No difference in CRP at ICU admission between survivors or nonsurvivors (ii) No decrease in CRP at day 3 was associated with a poor outcome	Same evolution for SOFA score but not for fever or leukocyte count

Vandijck et al. [26]	84 ICU patients with nosocomial bacteremia	Higher values of CRP with Gram-negative bacilli compared to Gram-positive cocci bacteremia	Review of the time course of CRP before the bacteremia Predictive factor?

Póvoa et al. [27]	44 ICU patients with bacteremia	CRP concentrations ratio start to change only at day 2 in survivors.	CRP ratio only predictive of outcome at day 4

Zhang and Ni [28]	Meta-analysis of 14 studies including 1969 patients	Evolution of CRP for more than 48 hours is predictive of outcome	Large heterogeneity of the studies (*I* ^2^ = 92%)

Póvoa et al. [29]	186 septic cancer patients with (*n* = 86) or without (*n* = 68) neutropenia	(i) CRP concentrations were higher in neutropenic patients (ii) No relation with the severity of the neutropenia	Same evolution of CRP between neutropenic and nonneutropenic patients

Fraunberger et al. [30]	38 ICU patients at the onset of fever	(i) Increase in CRP at the onset of fever (ii) No difference between survivors and nonsurvivors	Comparisons of CRP between ICU patients and volunteers

Su et al. [31]	144 ICU patients at the onset of fever (84 sepsis and 64 SIRS)	(i) CRP more elevated in septic compared to patients with SIRS (ii) CRP increase at the onset of fever and could discriminate patients with or without bacteremia	CRP concentrations were lower in patients with bacteremia

Christ-Crain et al. [32]	50 infected patients with (*n* = 24) or without appropriate antibiotics (*n* = 18) or peritonitis (*n* = 8)	An increase in CRP of at least 2.2 mg/dL in the first 48 h was associated with ineffective initial antibiotic therapy	(i) Only 8 patients with peritonitis (ii) No data on the timing of reintervention

Bota et al. [33]	864 patients with (*n* = 79) and without cirrhosis (*n* = 785)	(i) CRP levels were higher in cirrhotic with infection compared to cirrhotic patients without infection. (ii) No difference related to severity of the cirrhosis assessed by the Child-Pugh classification.	No data about CRP levels in relation with the severity of sepsis (SOFA, vasopressor dosage, PaO_2_/FiO_2_, extra renal replacement) for each level of cirrhosis

Silvestre et al. [34]	7 patients with hepatic failure	Low CRP levels in patients with infection	Few patients included. One with a diagnosis of hepatic failure at ICU day 26

**Table 2:** Summary of the studies in ICU patients with community-acquired (CAP) or ventilator-associated pneumonia (VAP).

Studies	Types of patients included	Conclusions	Remarks
Póvoa et al. [20]	48 patients with VAP	CRP levels were higher than in noninfected patients.	(i) No relationship with mortality was reported. (ii) No comparisons of CRP between patients with VAP compared to other infections

Hillas et al. [35]	45 patients with VAP	(i) No difference in CRP concentrations at VAP diagnosis between survivors and nonsurvivors (ii) Increase in CRP between days 1 and 7 increased the risk of developing septic shock	Long delay (7 days) for the diagnosis of inappropriate antibiotherapy

Seligman et al. [36]	75 patients with VAP	(i) No difference of CRP at admission between survivors and non survivors (ii) Decreased delta CRP between day 4 to 0 was associated with survival	No difference in outcome between patients with appropriate and inappropriate antibiotherapy

Póvoa et al. [37]	47 patients with VAP	By day 4, a CRP of 0.6 times the initial level was a marker of poor outcome	Importance of the CRP patterns at day 4 on outcome (fast response, nonresponse or biphasic response)

Coelho et al. [38]	53 patients with CAP	By day 3 a CRP level 0.5 times the initial level was a marker of poor outcome	Importance of the CRP patterns at day 3 on outcome (fast response, nonresponse or biphasic response)

Coelho et al. [39]	191 patients with CAP, 111 with mechanical ventilation	(i) No difference in CRP levels at admission between survivors and non-survivors (ii) CRP ratio (Day 7/Day 1) decreased significantly more rapidly in survivors	(i) Already at day 5, a CRP of above 0.5 of the baseline value was associated with a poor outcome. (ii) Same results for patients with CAP with mechanical ventilation.

Bajwa et al. [40]	177 patients with ARDS/ALI	(i) Lower CRP concentrations in non survivors compared to survivors (ii) Difference in CRP was observed in patients with pneumonia but not in trauma patients	Nonsurvivors who had a higher APACHE 3 score and were older and more cirrhotic were included in this group

Lisboa et al. [41]	68 ICU patients with VAP	Good correlation between the first bacterial load and CRP concentrations and between variations of bacterial load and CRP over time	(i) Relationship between bacterial burden and CRP (ii) A CRP ratio of 0.8 at 96 hours seems to be a useful indicator of adequate antibiotherapy

Bruns et al. [42]	289 patients with CAP, 137 with bacterial etiology	A decline of LESS than 60% in CRP levels in 3 days and a decline of LESS of 90% in CRP levels in 7 days were both associated with an increased risk of having received inappropriate empiric antibiotic treatment	Importance of the CRP patterns at days 3 and 5 on outcome (fast response, nonresponse, or biphasic response)

Menendez et al. [43]	658 patients with CAP	(i) CRP levels were higher in patients with CAP with an isolated microorganism than without (ii) CRP levels were significantly higher if CAP was associated with bacteremia (iii) No really great differences appears for CRP in relation to the type of causal microorganisms	Relation between causal microorganisms and CRP
